# Supplementary material for: Splice-Junction-Based Mapping of Alternative Isoforms in the Human Proteome
Source: Cell Rep. Author manuscript; Available in PMC 2020 Jan 15. (PMC6961840; doi:10.1016/j.celrep.2019.11.026)
Supplement: 2 [file NIHMS1546469-supplement-2.zip › DF1/PXD010154/Prostate/Prostate_18_SGSM3_NDIITELTPDYSMESHQR.pdf]

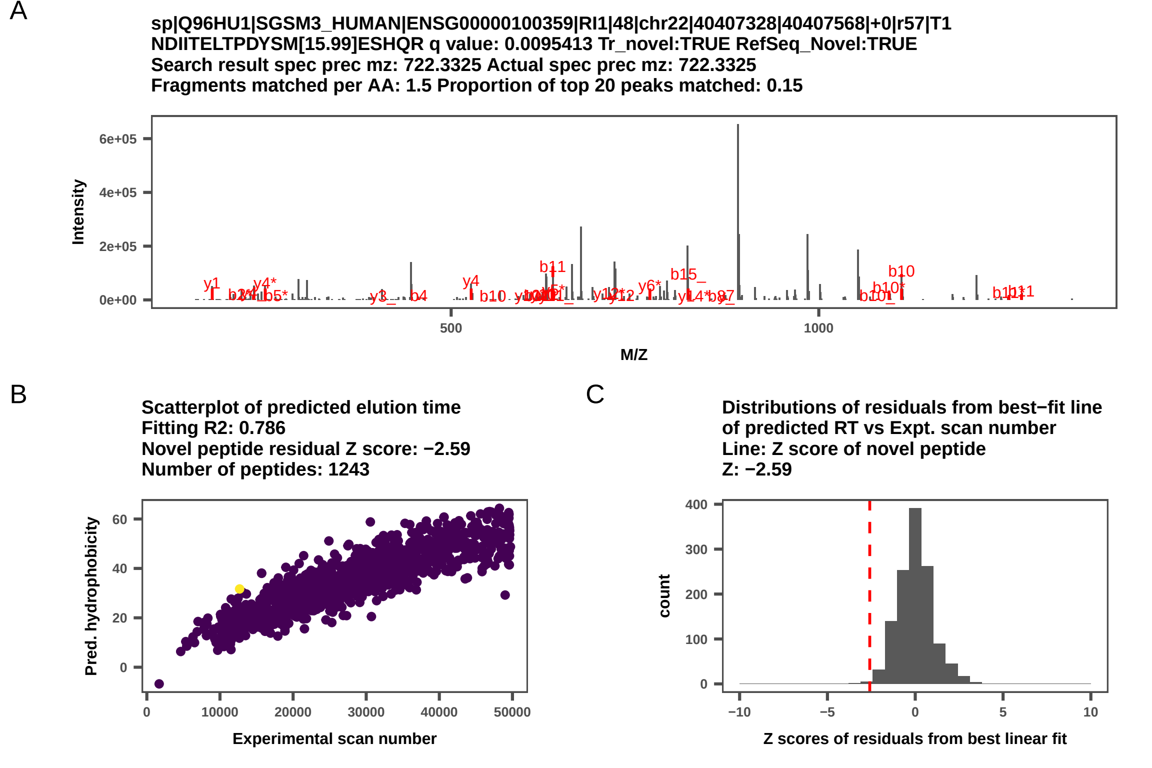

A
sp|Q96HU1|SGSM3_HUMAN|ENSG00000100359|RI1|48|chr22|40407328|40407568|+0|r57|T1
NDIITELTPDYSM[15.99]ESHQR q value: 0.0095413 Tr_novel:TRUE RefSeq_Novel:TRUE
Search result spec prec mz: 722.3325 Actual spec prec mz: 722.3325
Fragments matched per AA: 1.5 Proportion of top 20 peaks matched: 0.15
Intensity
6e+05
4e+05
2e+05
0e+00
500
1000
M/Z
y1
y4*
b5*
y3_
b4
y4
b10
b11
b15_
y6*
b10
b10*
b10_
B
Scatterplot of predicted elution time
Fitting R2: 0.786
Novel peptide residual Z score: −2.59
Number of peptides: 1243
Pred. hydrophobicity
60
40
20
0
0
10000
20000
30000
40000
50000
Experimental scan number
C
Distributions of residuals from best−fit line
of predicted RT vs Expt. scan number
Line: Z score of novel peptide
Z: −2.59
count
400
300
200
100
0
−10
−5
0
5
10
Z scores of residuals from best linear fit
